# Computational Model of Progression to Multiple Myeloma Identifies Optimum Screening Strategies

**DOI:** 10.1200/CCI.17.00131

**Published:** 2018-03-22

**Authors:** Philipp M. Altrock, Jeremy Ferlic, Tobias Galla, Michael H. Tomasson, Franziska Michor

**Affiliations:** **Philipp M. Altrock**, Moffitt Cancer Center and Research Institute; Morsani College of Medicine, University of South Florida, Tampa, FL; **Jeremy Ferlic** and **Franziska Michor**, Dana-Farber Cancer Institute and Harvard University; Harvard T.H. Chan School of Public Health, Boston; **Franziska Michor**, Center for Cancer Evolution, Dana-Farber Cancer Institute, and The Ludwig Center at Harvard, Boston; Broad Institute of Harvard and Massachusetts Institute of Technology, Cambridge, MA; **Tobias Galla**, University of Manchester, Manchester, United Kingdom; and **Michael H. Tomasson**, Roy J. and Lucille A. Carver College of Medicine, University of Iowa, Iowa City, IA.

## Abstract

**Purpose:**

Recent advances have uncovered therapeutic interventions that might reduce the risk of progression of premalignant diagnoses, such as monoclonal gammopathy of undetermined significance (MGUS) to multiple myeloma (MM). It remains unclear how to best screen populations at risk and how to evaluate the ability of these interventions to reduce disease prevalence and mortality at the population level. To address these questions, we developed a computational modeling framework.

**Materials and Methods:**

We used individual-based computational modeling of MGUS incidence and progression across a population of diverse individuals to determine best screening strategies in terms of screening start, intervals, and risk-group specificity. Inputs were life tables, MGUS incidence, and baseline MM survival. We measured MM-specific mortality and MM prevalence after MGUS detection from simulations and mathematic modeling predictions.

**Results:**

Our framework is applicable to a wide spectrum of screening and intervention scenarios, including variation of the baseline MGUS to MM progression rate and evolving MGUS, in which progression increases over time. Given the currently available point estimate of progression risk reduction to 61% risk, starting screening at age 55 years and performing follow-up screening every 6 years reduced total MM prevalence by 19%. The same reduction could be achieved with starting screening at age 65 years and performing follow-up screening every 2 years. A 40% progression risk reduction per patient with MGUS per year would reduce MM-specific mortality by 40%. Specifically, screening onset age and screening frequency can change disease prevalence, and progression risk reduction changes both prevalence and disease-specific mortality. Screening would generally be favorable in high-risk individuals.

**Conclusion:**

Screening efforts should focus on specifically identified groups with high lifetime risk of MGUS, for which screening benefits can be significant. Screening low-risk individuals with MGUS would require improved preventions.

## INTRODUCTION

Multiple myeloma (MM) is the second most common hematologic malignancy in the United States, representing 1.8% of new cancer cases and 2.1% of deaths resulting from annually.^1^ MM is an incurable plasma-cell malignancy.^2^ Patients show abnormal levels of the paraprotein M protein,^3^ indicating a monoclonal cell population and end-organ damage such as lytic bone lesions.^4^ Almost all patients with MM experience progression from a precursor condition called monoclonal gammopathy of undetermined significance (MGUS), displaying only M protein spikes.^4^ The MGUS condition exists in approximately 2% of the population age ≥ 50 years.^5^ Men show higher age-adjusted incidence rates than women.^6^ There are also racial disparities; MGUS prevalence in African Americans age 40 years is roughly equivalent to MGUS prevalence in non-African Americans age 50 years.^7^

Recent advances suggest that the rate of progression to MM can be altered by therapeutic interventions.^8,9^ Obesity—a modifiable risk factor for MM—is associated with increased risk.^10-12^ Furthermore, metformin is associated with a reduced progression of MGUS to MM, potentially delaying MM by 4 years in patients with type 2 diabetes with MGUS.^9^ Reduced risk is also associated with regular use of aspirin.^8^ Although causal relationships and molecular mechanisms of these associations are uncertain, these findings suggest that pharmacologic and other interventions have the potential to reduce the risk of MGUS progression. It is therefore of particular interest to investigate the effects of screening for MGUS, especially in specific subpopulations, and screening distribution across risk groups. The goals of screening are to detect MGUS early and reduce MM prevalence and mortality as a result of mild interventions leading to an MGUS to MM progression risk reduction.

Independent of intervention-based progression risk reduction, precursor state knowledge can also affect mortality and comorbidity in patient cohorts. Sigurdardottir et al^13^ found that patients with MM with prior knowledge of MGUS had improved overall survival (median, 2.8 years) compared with patients with MM without prior knowledge (median, 2.1 years), overshadowed by a larger extent of relative comorbidities in patients with prior knowledge. The authors concluded that earlier treatment of MM, as a result of prior knowledge, leads to better survival (potentially conflicted by lead bias). Clinical follow-up in cases of accidental MGUS detection may be important regardless of (anticipated) risk type,^13^ and follow-up preceding the diagnosis of MGUS-associated malignancy may lead to improved survival.^14^ Screening for MGUS might have additional merit because < 10% of MM diagnoses currently are knowingly associated with preexisting MGUS.^13,14^

We designed a computational model that describes incidence of MGUS and progression to MM, specific MGUS screening scenarios, and potential epidemiologic changes, implemented after detection. Our model is based on life tables and epidemiologic data of MGUS and MM, which depend on genetic background, sex, and age^15,16^ and correlate with ethnicity.^17^ Using simulations and analytic results, we assessed whether a given reduction in progression risk after a positive MGUS screen could reduce MM prevalence and lead to changes in MM-specific mortality (or survival). Our work can be used to identify optimal screening strategies and can assess the utility of interventions targeting MM precursor states.

## MATERIALS AND METHODS

**Fig 1.**
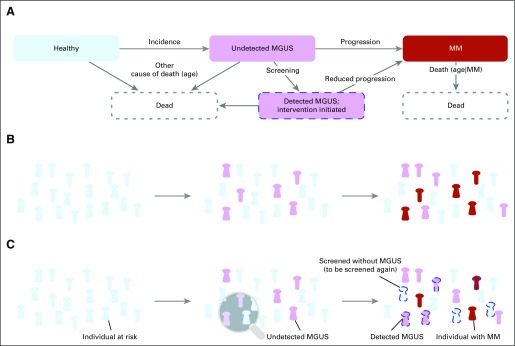
Population dynamics of unscreened and screened individuals with monoclonal gammopathy of undetermined significance (MGUS) as well as those with multiple myeloma (MM). (A) Possible individual transitions from healthy to MGUS to MM can be modeled as a Markov chain. The transitions describe incidence and screening of MGUS and progression to MM. The four possible states are healthy (blue), undetected MGUS (pink), detected MGUS (pink with dashed outline), and MM (red). (B) Example time evolution of a cohort at risk for MGUS and subsequent MM without screening. Undetected MGUS cases accumulate and can lead to a baseline number of MM cases. (C) Time evolution of a cohort with screening and intervention that reduces MGUS to MM progression. MGUS cases accumulate; individuals are screened and receive preventive treatment if positive for MGUS, leading to a lower number of MM cases (red indicates a few screened individuals who may develop MM nonetheless).

We developed a Markov chain model (Fig 1A) in which healthy individuals transition to an undetected MGUS stage, from which they can transition to detected MGUS if screened. An individual with MGUS progresses to overt MM at a certain rate per year; however, a positive MGUS screening result reduces the rate of progression to MM (Figs 1B and 1C). Individuals may die at any point, but mortality is greater for those with MM. We performed stochastic simulations and derived an analytic framework to assess MM mortality and prevalence reduction after screening (Data Supplement).

### Model Inputs and Outputs

We were interested in screening outcomes in mixture populations composed of individuals with different MGUS lifetime risks. We distinguished non-African American and African Americans as low-risk (baseline) and high-risk individuals, respectively. From baseline, high-risk individuals carry an average two-fold increase in lifetime risk of MGUS.^16,18^ Calculations of the respective MGUS incidence rates are displayed in the Data Supplement. Furthermore, we used a crude birth rate for the total population and life tables to calculate death events of healthy individuals and those with MGUS (high- and low-risk men and women), MM-specific death rates, and a fixed MGUS to MM progression rate for unscreened individuals. A screening scenario was specified by three parameters: age of the individual when receiving the first screen (*a_0_*), spacing between follow-up screens (Δ*a*), and risk reduction *r* after a positive screen (Table 1). As model outputs, we were interested in the effects of varying screening scenarios on MM-specific mortality after MGUS detection and on the fraction of individuals with MM of all ages. We initiated all simulated populations according to the age distribution of the population in the United States according to the 2013 census,^19,20^ with a fixed fraction of healthy high-risk individuals of 20%. Although the fraction of African Americans in the United States is approximately 13%,^19^ we estimated that the genetic diversity in the United States would further contribute to high risk.

**Table 1.**
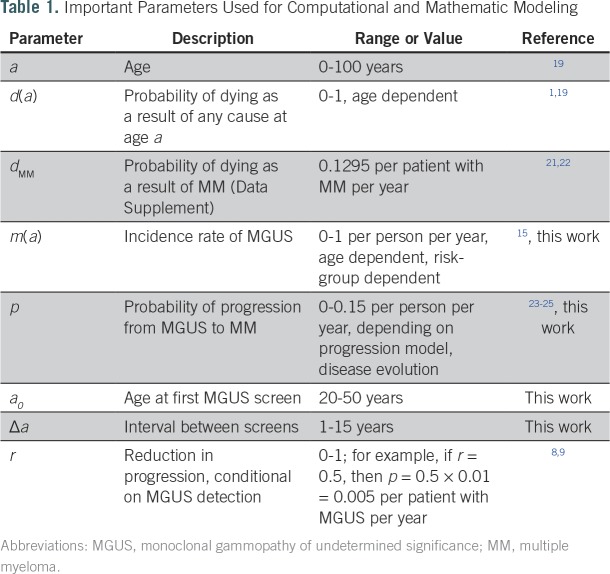
Important Parameters Used for Computational and Mathematic Modeling

### Stochastic Model

We simulated the Markov chain model (Fig 1A; Data Supplement) by using a fixed crude birth rate,^26^ age-dependent death rates for healthy individuals and those with MGUS individuals,^19^ and a fixed death rate for patients with MM.^27^ From the baseline low-risk MGUS incidence adapted from Therneau et al,^15^ we calculated elevated incidence rates per life-year for specific risk groups. In our simulations, high-risk African Americans experience MGUS incidence that exponentially increases with age such that lifetime risk is approximately two-fold higher than that at baseline (low risk).^16,28^ Progression to MM was mostly constant across risk groups^23^ and occurred at a rate of *p* = .01 per year in MGUS-positive but unscreened individuals.^24^ Screening meant that starting at age *a_0_*, individuals were screened each year with probability 1/Δ*a*, such that their average time between screens was Δ*a*. Positively screened individuals were assumed to experience progression at a reduced rate of *r* × *p*. Recent studies have estimated *r* = 0.61 for regular aspirin users.^8^ From simulations, individual ages, MGUS status, MGUS screening, and MM status were recorded (Data Supplement). This approach allowed us to calculate MGUS and MM prevalence, distribution of age at diagnosis of MM, and MM-specific mortality. We also devised a model to calculate MGUS and MM prevalence and mortality analytically (Data Supplement). Using this framework, we calculated the fractions of individuals with MGUS *M* at a specific age for any risk group, the fraction of individuals with MM proportional to *M*, and the MM-specific mortality for a given number of years after MGUS detection.

## RESULTS

### Prevalence of MM When Screening for MGUS

**Fig 2.**
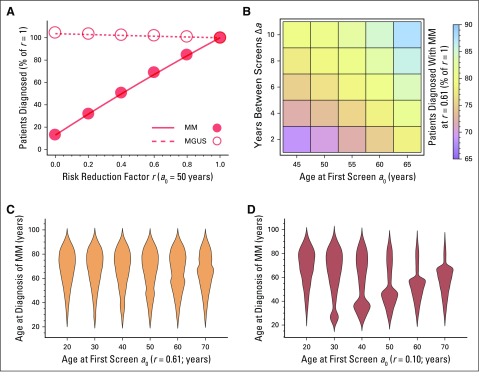
Number of patients with multiple myeloma (MM), age at MM diagnosis, and variability of screening strategy. (A) When monoclonal gammopathy of undetermined significance (MGUS) screening was applied, we measured the number of patients diagnosed with MGUS (dashed line, open circles) and MM (solid line, filled circles) relative to the *r* = 1 values, with respect to changing the risk reduction factor *r* (circles, simulations; lines, analytic model; Data Supplement), with *a*_0_ = 50 years and Δ*a* = 1 year. At *r* = 0.61, the MM fraction dropped to < 70% of its value at *r* = 1 (where screening had no effect on progression). (B) Variability in MM fraction at *r* = 0.61, with respect to changes in *a*_0_ and Δ*a* (analytic approach, point estimates; Table S4, Data Supplement). (C, D) Distributions of age at MM diagnosis (Δ*a* = 1 year), with varying *a*_0_ and fixed *r* of (C) 0.61 or (D) 0.1. Width in these violin plots is equal to probability of MM diagnosis at that age. All point estimates were calculated from a simulation of approximately 10^8^ individuals.

We performed stochastic simulations of our agent-based model to investigate the effects of different conditions on MGUS and MM prevalence and mortality. As expected, the proportions of individuals with MGUS and MM varied with the fraction of high-risk persons in the population (Data Supplement). An increasing risk reduction after a positive MGUS screen drastically diminished the fraction of patients with MM while increasing the fraction of those with MGUS (Fig 2A). To validate our results, we compared our findings with those of Birmann et al,^8^ where in a cohort of 163,810 men and women, 82 individuals were associated with the baseline progression risk and 44 were associated with the lowest progression risk measured, with a value of *r* = 0.61 in long-term aspirin users (95% CI, 0.41 to 0.95). Birmann et al reported a reduction linked to aspirin use of 40% in patients with MM. On the basis of this study, we estimated a reduced risk in progression from MGUS to MM of *r* = 0.61 (point estimate). For this value, our predictions of approximately 60% lie in the CI of Birmann et al for *r*.

Changes in onset age of screening *a*_0_ and spacing Δ*a* affected MM risk reduction similarly (Fig 2B; Data Supplement). For example, for a fixed *r* = 0.61, *a*_0_ = 45 years and Δ*a* = 8 years reduced MM prevalence to 77.2%, whereas *a*_0_ = 65 years and Δ*a* = 8 years reduced MM prevalence to 78.6% relative to *r* = 1, respectively. Even for nearly complete risk reduction (*r* close to 0) and rare screening (Δ*a* = 8 years), *a*_0_ = 45 years reduced cases of MM by 60% and *a*_0_ = 65 years by approximately 38%. Figures 2C to 2F show the impact of Δ*a* and *a*_0_ on the age distribution of MM diagnoses, varying *r*. These normalized violin plots give the probability of finding an individual of a specific age with MM in our simulations. The bottleneck near *a*_0_ is more pronounced for lower *r* values. Hence, both the number of cases of MM and age at MM diagnosis are sensitive to changes in progression risk, screening interval, and screening start age.

### Lead-Time Bias and Cumulative MM-Specific Mortality

**Fig 3.**
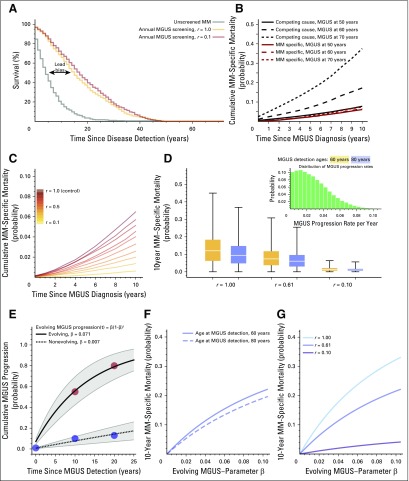
Lead-time bias, cumulative multiple myeloma (MM) –specific mortality, and monoclonal gammopathy of undetermined significance (MGUS) to MM progression variability. All simulations were performed with populations of 10^8^ healthy individuals (20% high risk). (A) Potential lead-time bias, comparing median survival after MM diagnosis without screening (blue: median survival, 4 years) and with screening (gold: median survival, 15 years; gray: median survival, 17 years after MGUS screen, respectively). Without screening, disease detection was the event of MM diagnosis. With screening, disease detection was diagnosis of asymptomatic MGUS. (B) Cumulative MM-specific mortality in years after MGUS detection was measured for the groups of 50, 60, and 70 years of age at MGUS detection (*a*_0_ = 50 years, Δ*a* = 1, and *r* = 1). In older patients, death resulting from other cause becomes more dominant. (C) MM-specific mortality changed dramatically with *r* (*a*_0_ = 50 years, Δ*a* = 1), here shown for individuals diagnosed with MGUS at age 60 years, sampled from simulations. (D) MM-specific mortality is influenced by variability in MGUS to MM progression rate^23^ (inset, truncated normal distribution\; mean, 0.01; standard deviation, 0.03), for different *r*, using the analytic model (Δ*a* = 1; Data Supplement). (E) Simple evolving MGUS progression rates [β × (1 − β)*^t^*], fitted to data from Rosiñol et al^25^ (filled circles; nonevolving: 10% at 10 years, 13% at 20 years follow-up; evolving: 55% at 10 years, 80% at 20 years follow-up), for which we show 95% CIs. Nonevolving MGUS confirms the low value of β (here 0.007; *R*^2^ = 0.996), corresponding to constant progression risk *p* (Table 1). Evolving MGUS led to a progression rate of *p* = .071 (*R*^2^ = 0.975). (F, G) Impacts of age at MGUS detection and progression risk reduction *r* on MM-specific mortality as a function of evolving progression rate calculated as described in Data Supplement: (F) *r* = 0.61 and (G) age at MGUS detection 60 years.

Screening can cause lead-time bias; the survival time after a positive MGUS screening outcome is typically longer than the survival time after direct clinical presentation of MM, with or without screening; the difference between these two times is the lead-time bias.^29,30^ Because lead-time bias overshadows actual survival benefits of screening in clinical settings where this time difference may not be directly observed, disease-specific mortality is a more appropriate measure.^31^ We determined the expected lead-time bias by a comparison of survival in unscreened (control) and screened population simulations (Fig 3A). Median survival after MM diagnosis in the control group was 4 to 5 years. Median survival after MGUS detection (*a*_0_ = 50 years; Δ*a* = 1 year) was 15 years for *r* = 1.0 (and similar for *r* = 0.61) and 17 years for *r* = 0.1. Thus, the lead-time bias here would be 10 years.

We calculated the cumulative MM-specific mortality after MGUS detection, defined as the probability that an individual would die as a result of MM within a predefined number of years after detection of MGUS at a fixed age.^32^ We distinguished death events resulting from MM and deaths resulting from other causes. In Figure 3B, we display the MM-specific mortality as well as competing risk for MGUS detection at ages 50, 60, and 70 years. In younger groups, the chance of dying as a result of MM was comparable to the chance of dying as a result of other causes; the latter increased with age. MM-specific mortality varied strongly with the risk reduction factor *r* (Fig 3C). As shown, using the analytic model in the Data Supplement, MM-specific mortality should not be affected by the screening parameters *a*_0_ and Δ*a*, which only determine age-specific prevalences.

### MGUS to MM Progression Variability and Evolving MGUS

Our framework allows assessment of the impact of variation in MGUS progression rates,^23^ as well as the impact of evolving MGUS,^25^ in which the progression rate changes over time. Variability in MGUS progression rate *p* (per individual per year) can lead to large variability in mortality 10 years after MGUS detection if screening has no effect (*r* = 1.0), but this effect is reduced as risk reduction takes effect (*r* < 1; Fig 3C).

Patients with MGUS belong either to a large group of individuals who experience progression at a constant rate or to a small group who experience progression at an accelerating rate.^25^ Of 359 cases of MGUS reported by Rosiñol et al,^25^ 330 (92%) were nonevolving and 29 (8%) were evolving (Fig 3E). We approached this effect by assuming that for each individual, the rate to progress after exactly *t* years was given by the β × (1 − β)*^t^* (Fig 3; Data Supplement). We inferred that individuals with nonevolving MGUS experience progression at β = 0.007, which well approximates our constant progression rate of *p* = 0.01. Individuals with evolving MGUS experience progression with a 10-fold higher value (β = 0.07). MM-specific mortality increases considerably with evolving MGUS rate (Fig 3F) and decreases with *r* (Fig 3G). In addition to population-based diversity, global migration could affect the value of screening,^33^ as discussed in the Data Supplement using data from Ghana.^18^ Realistic levels of immigration of high-risk individuals are unlikely to affect US MGUS or MM statistics (Data Supplement).

### Equal Reduction of MM Prevalence Can Serve As a Criterion for Optimal Screening Frequency Among High- and Low-Risk Populations

**Fig 4.**
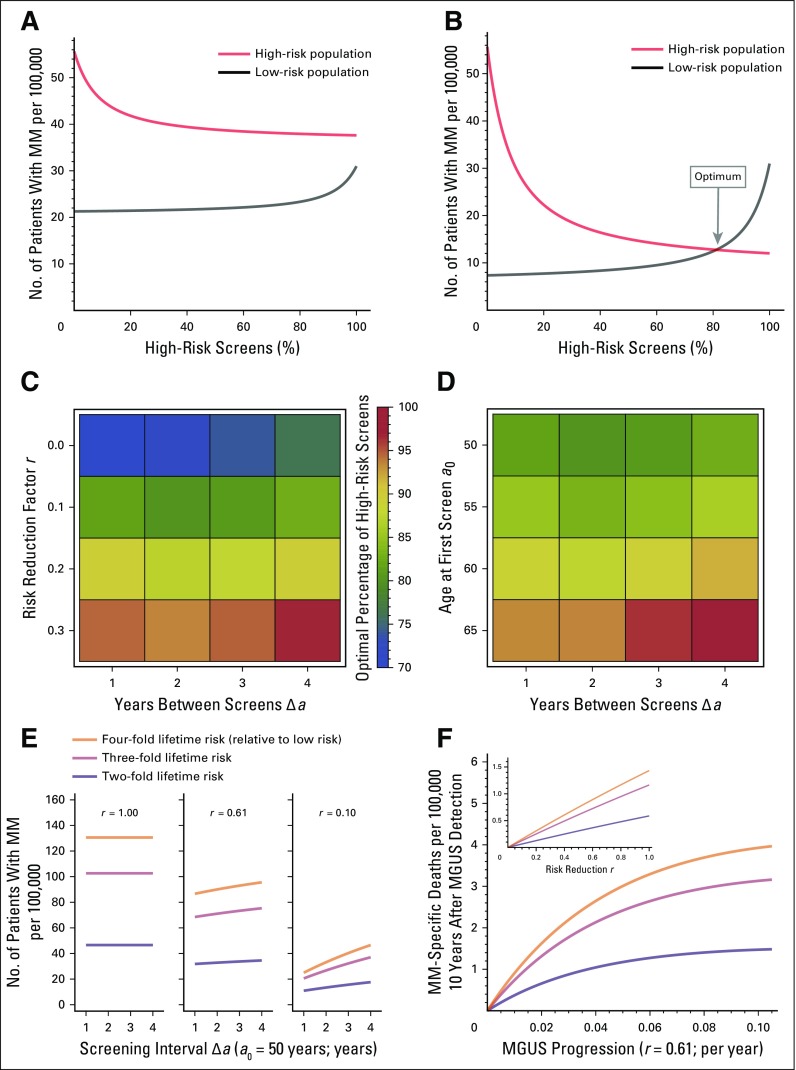
Equal disease fractions as a criterion for optimal screening distribution. (A, B) Comparing multiple myeloma (MM) fractions in the high-risk and low-risk populations (men and women, respectively), with *a*_0_ = 50 years and Δ*a* = 1 year, for different *r*. (A) For *r* = 0.61, equality could not be observed for any percentage of high-risk screens. (B) For *r* = 0.1, equality was observed at approximately 81% high-risk screens. Thus, an optimal fraction of screens was defined as the point where the fractions of patients with MM in both subpopulations were the same. (C) Location of the optimal fraction (scale) under variation of *r* and Δ*a* (Table S5, Data Supplement), with *a*_0_ = 50 years. Changing *r* from 0 to 0.3 would lead to up to 20% change in the optimal high-risk fraction of screens. Changing Δ*a* from 1 to 4 would lead to 1% to 3% change in the optimal high-risk fraction of screens. (D) For fixed *r* = 0.1, changes in *a*_0_ had more drastic effects than changes in Δ*a* (Table S6, Data Supplement). (E) For risk groups with a lifetime risk higher than two-fold, we examined the effect of risk reduction and screening interval (*a*_0_ = 50 years) on the number of patients with MM (Data Supplement). (F) MM-specific deaths per 100,00 were calculated as the product of screened individuals with monoclonal gammopathy of undetermined significance (MGUS) at age 60 years and the 10-year follow-up MM-specific mortality (*a*_0_ = 50 years and Δ*a* = 1; age at MGUS detection, 60 years). Both risk reduction and spacing of screens have more pronounced effects in higher-risk groups.

We sought to identify best screening distributions among different risk groups to minimize MM prevalence (Data Supplement). A fraction *y* of available screenings could be applied to high-risk individuals and the remainder, 1 − *y*, to low-risk individuals. There can exist a value of *y* for which MM prevalences are equal. If *r* = 1, no intercept exists, and all screening efforts would go to high-risk individuals (Fig 4A). The point estimate *r* = 0.61^8^ also gave *y* = 1. Lower values of *r* could permit values of *y* < 1 (Fig 4B), ranging from *y* = 71% (*r* = 0.0) to *y* = 96% (*r* = 0.3), given *a*_0_ = 50 years (Fig 4C; Data Supplement); *y* was between 81% and 93% for Δ*a* = 1 and between 79% and 95% for Δ*a* = 4 (fixed *r* = 0.1; Fig 4D; Data Supplement).

### Groups With Higher Than Two-Fold Lifetime Risk Could Bnefit Strongly From Regular Screening

Multiple factors determine increased lifetime risk of MGUS, notably family history of MM.^34^ We analyzed the sensitivity of MM prevalence and MM-specific mortality to screening frequency and risk reduction. Both risk reduction and spacing of screens have more pronounced effects in higher-risk groups, but in those groups, steeper increase in mortality was observed with decreasing screening frequency (Fig 4E). Importantly, the increase in MM-specific deaths saturated with increasing progression rate, indicating that in high-risk groups, mortality reduction can be achieved in subgroups of intermediate progression rates (Fig 4F).

## DISCUSSION

MM remains incurable for a majority of patients, and decreasing mortality is of as much interest as decreasing its prevalence.^11^ All patients seem to experience progression to symptomatic MM from a premalignant, asymptomatic stage called MGUS.^35^ The fact that there are outstanding diagnostic tests for MGUS implies the possibility of delaying progression of MGUS to MM by screening and early identification.^36^ Because precise estimates of MGUS prevalence have changed over the past decade,^5-7,37^ we considered relative changes in prevalence (using as a baseline no effect of screening on progression risk reduction). We evaluated a range of possible screening strategies based on the consideration that diagnosis of MGUS permits progression reduction as a result of several possible interventions or modifiable risk factors, including aspirin, metformin, or mediation such as exercise or diet alterations.^8,9,11,12,36^

The promise of early intervention in MGUS should be viewed with caution. Our current understanding comes from retrospective observational studies. Our results, however, suggest that research to identify effective chemoprevention agents in high-risk MGUS can be justified. It will take time to develop a more comprehensive understanding of the intricate relationship between early intervention utilities and potential adverse effects on a wider scale, related to health care costs and psychological burden. Patients with MGUS may experience psychological distress similar to that experienced by those with MM, and the identification of cancer precursor states must be accompanied by a discussion of the utility of follow-up in individual patients.^38-42^ Promising efforts that evaluate MGUS screening and continuous follow-up before clinical manifestation of MM are under way in a long-term, prospective, three-armed randomized trial (iStopMM).^43^ Such long-term efforts highlight the utility of predictive tools such as the one developed here.

Our approach allowed us to quantify the amount of risk reduction needed to result in certain reductions in MM-specific mortality and MM prevalence (measured as MM fraction). To avoid lead-time bias, we evaluated screening scenarios in terms of mortality and MM prevalence. Length-time bias, in contrast, is a form of selection bias that occurs because of heterogeneity in the progression speed of a malignancy. This bias was absent in our study because we modeled uniform progression of the disease (ie, a high-risk person with early incidence of MGUS experienced progression to MM equally as fast as a low-risk person with late MGUS incidence; the time spent in the MGUS state in the no-screening scenario was independent of age).^16^ Therefore, these common sources of bias in epidemiologic prevention studies did not confound our results.

Using a stochastic simulation framework and an analytic model, we measured MGUS and MM prevalence and MM-specific mortality in different risk groups for different screening strategies and varying progression risk reduction after MGUS detection. For effective MM prevalence reduction, better screening results are expected for screening as early as possible and frequent follow-up. Improved chemoprevention, effectively reducing progression risk, may also reduce MM-specific mortality. We found that this effect is more pronounced in individuals with evolving MGUS, especially in individuals with higher than two-fold lifetime MGUS risk.

We did not explicitly address screening toxicity, nor did we model smoldering MM—an intermediate stage between MGUS and MM with a much higher rate of progression to full MM of approximately 30% per year—in part because it remains unclear whether smoldering MM is a requisite intermediate between MGUS and MM. However, our framework can be adjusted and expanded.

Assessments of screening and prevention in solid tumors (eg, prostate cancer) have been controversial and lacking in evidence for screening in large prospective trials.^44^ We share the skepticism of potential medicalization of asymptomatic conditions. However, the biology of MGUS and the robust laboratory tests demand careful evaluation of the role of screening and prevention. With notable similarities in the epidemiology of prostate cancer and MGUS (ie, most low-grade lesions will not proceed to lethal disease), major differences in technology of screening tests for these diseases are critical. Prostate-specific antigen tests for prostate cancer are burdened by substantial false-positive (21% to 32% sensitivity) and false-negative rates (85% to 91% specificity).^45^ In contrast, serum testing for MGUS is straightforward. The sensitivity of serum protein electrophoresis and free light chain testing for MGUS is close to 100%, and the specificity is 99%.^46^ These differences underline the evaluation of the role of screening and prevention in MGUS and MM. We have shown that the reduction of cases of MM and MM-specific mortality in high- and low-risk subpopulations can be achieved, but only for drastic reduction in progression risk. Until highly effective agents are developed, identification and follow-up of high-risk individuals are important. Screening for MGUS may have significant population benefits by lowering the incidence of MM, provided effective and nontoxic interventions can be identified. Without further study of chemoprevention strategies, regular screening of MGUS candidates should start as early as possible, with biannual follow-up, and focus on high-risk individuals, especially those with a family history of MM, or on groups with strong indication for evolving MGUS progression.
